# 67/w mit Herz-Kreislauf-Stillstand: Vorbereitung auf die Zusatz-Weiterbildung Internistische Intensivmedizin: Fall 9

**DOI:** 10.1007/s00063-026-01420-x

**Published:** 2026-03-17

**Authors:** Friederike Bennett, Tobias Wengenmayer

**Affiliations:** 1 UCL Medical School, Research Department of Medical Education (RDME), University College London, 40 Bernard Street, WC1N 1LE London, Großbritannien; 2 Interdisziplinäre Medizinische Intensivtherapie (IMIT), Universitätsklinikum Freiburg, 79106 Freiburg, Deutschland

**Keywords:** Kreislaufstillstand, Reanimation, Ethische Aspekte, Postreanimationsbehandlung, Neurologische Prognose

## Prüfungssimulation

### Fallschilderung

**Abb. 1:**
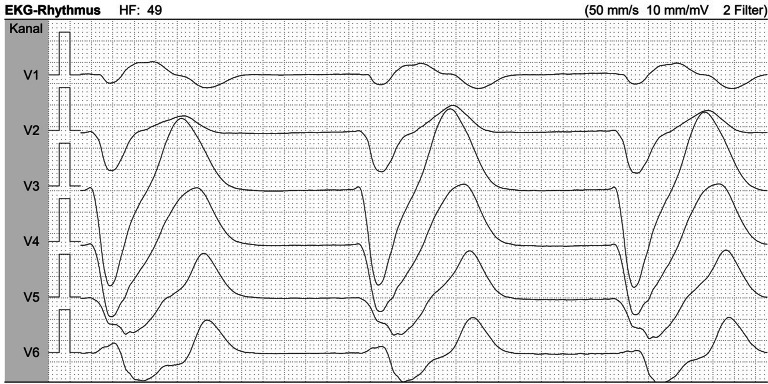
Initialer EKG-Rhythmus der Patientin. *EKG* Elektrokardiogramm, *HF* Herzfrequenz

Sie beginnen den Tagdienst auf der Intensivstation, als das Reanimationstelefon klingelt. Gemeinsam mit einer Intensivpflegekraft werden Sie auf die pneumologische Station gerufen. Vor Ort laufen bereits adäquate Basismaßnahmen der Reanimation mit einem Verhältnis Herzdruckmassage zu Beutel-Masken-Beatmung 30:2. Während Ihr Teammitglied den Defibrillator anschließt, erfahren Sie: Die 67-jährige Patientin wurde in der Nacht mit Dyspnoe aufgenommen; der Kollaps wurde von der Bettnachbarin beobachtet. Nach Anschluss des Defibrillators führen Sie eine sofortige Rhythmusanalyse durch (Abb. 1). Die Patientin ist weiterhin pulslos und apnoisch.

## Prüfungsfragen

- Welchen initialen Rhythmus erkennen Sie und wie führen Sie die Reanimation leitliniengerecht fort?
- Wann setzen Sie mechanische Thoraxkompressionsgeräte ein und in welchen Situationen erwägen Sie eine extrakorporale kardiopulmonale Reanimation?
- Was sollten Sie hinsichtlich des Atemwegsmanagements während der Reanimation beachten?
- Welche reversiblen Ursachen müssen Sie berücksichtigen und wie können diese behandelt werden?
- Welche Maßnahme hätte den Kreislaufstillstand möglicherweise verhindern können?
- Nach welchen Kriterien entscheiden Sie, ob die Reanimation fortgeführt oder beendet wird?
- Welche Maßnahmen gehören zur strukturierten Postreanimationsbehandlung nach Wiedereintritt eines Spontankreislaufs?
- Wie beurteilen und managen Sie die neurologische Prognose im weiteren Verlauf?

### Antworten

#### Welchen initialen Rhythmus erkennen Sie und wie führen Sie die Reanimation leitliniengerecht fort?

Es handelt sich um eine **pulslose elektrische Aktivität** (PEA):

- Elektrische Aktivität ist sichtbar, jedoch ohne tastbaren Puls und ohne hämodynamische Wirkung – somit besteht **weiterhin ein Kreislaufstillstand**.
- Der Großteil (> 85 %) der innerklinischen Kreislaufstillstände weist initial einen **nichtdefibrillierbaren Rhythmus** auf [1].

Bei einem nichtschockbaren Rhythmus sollten nach den **Leitlinien des European Resuscitation Council** (ERC; [2]) jetzt folgende Maßnahmen eingeleitet werden:

- **Sofortige Fortsetzung der Reanimation für 2** **min bis zur nächsten Rhythmuskontrolle**
- Hier sollte auf Folgendes geachtet werden:
  - Thoraxkompressionen und Beatmung weiterhin im Verhältnis 30:2 bis zur Atemwegssicherung.
  - Qualitativ hochwertige Thoraxkompressionen (Druckpunkt: untere Hälfte des Brustbeins; Drucktiefe: 5–6 cm; Frequenz: 100–120/min, vollständige Entlastung).
  - Minimale Unterbrechung der Kompressionen und Helferwechsel alle 2 min.
  - Gabe von 100 % O_2_.
- **Etablierung eines i.v.-Zugangs zur Medikamentengabe, ggf. i.o.-Zugang erwägen**
- **Sofortige Gabe von 1** **mg Adrenalin i.v./i.o.** nach Etablierung eines Zugangs, **wiederholte Gabe alle 3–5** **min**

#### Wann setzen Sie mechanische Thoraxkompressionsgeräte ein und in welchen Situationen erwägen Sie eine extrakorporale kardiopulmonale Reanimation?

- **Mechanische Geräte zur Thoraxkompression** sollten nicht routinemäßig und sofort angewendet werden, sondern nur wenn
  - **manuelle Kompressionen nicht praktikabel** sind,
  - die Kompressionen ein **Sicherheitsrisiko für den Anwender** darstellen oder
  - eine **Ermüdung** droht.
- Zusammengefasst kann der Einsatz in folgenden Situationen erwogen werden:
  - **Bei länger anhaltenden Reanimationen**, z. B. während eines Transports, unter Therapie bei Hyperkaliämie oder unter Thrombolyse bei Lungenarterienembolie.
  - **Periinterventionell oder während diagnostischer Maßnahmen** (z. B. perkutane Koronarintervention [PCI], Computertomographie [CT]) und **während des innerklinischen Transports**.
  - Zur Aufrechterhaltung des Kreislaufs im Rahmen einer **Organspende**.
  - **Überbrückend vor extrakorporaler kardiopulmonaler Reanimation** (eCPR).

##### Cave.

Anwender sollten bzgl. der Anwendung der Geräte geschult sein.

- **Der Einsatz einer eCPR** [2] kann u. a. **zur zeitlichen Überbrückung bei bestehenden reversiblen Ursachen des Herz-Kreislauf-Stillstands** angewendet werden, z. B.
  - bei **refraktärem Kammerflimmern**,
  - **periinterventionell **(z. B. PCI, Thrombektomie),
  - bei **Hypothermie**.
- **Folgende Kriterien sollten zudem erfüllt sein:**
  - Unmittelbarer Beginn der Reanimation nach beobachtetem Kreislaufstillstand.
  - Patienten ≤ 75 Jahre, geringe/keine Komorbidität.
- **Weitere Kriterien**, bspw. der initiale Rhythmus oder der Wert des endtidalen Kohlendioxids (etCO_2_), können zur Entscheidungsfindung herangezogen werden. Im Zweifel sollte deshalb bzgl. der Indikationsstellung **Rücksprache mit einem Zentrum für eCPR** gehalten werden.

#### Was sollten Sie hinsichtlich des Atemwegsmanagements während der Reanimation beachten?

- Nach den ERC-Leitlinien sollte das Atemwegsmanagement **angepasst an die aktuelle Situation, Ressourcen und Priorität von Aufgaben schrittweise** erfolgen.
- Ziel ist eine **effektive Beatmung mit Gabe von 100** **% O**_**2**_** unter Reanimation**.
- Ein **erweitertes Atemwegsmanagement** kann demzufolge **ggf. zu einem späteren Zeitpunkt** erfolgen, solange einfache Maßnahmen effektiv sind.
- **Zudem ist Folgendes zu beachten:**
  - Das Atemwegsmanagement ist delegierbar.
  - Die endotracheale Intubation sollten jedoch nur erfahrene Anwender durchführen.
  - Nutzung von Videolaryngoskopie erwägen.
  - Bestätigung der Position des Endotrachealtubus mittels Kapnographie.
  - Nutzung von alternativen Atemwegshilfen (z. B. Larynxmasken) erwägen.
  - Ein erweitertes Atemwegsmanagement ermöglicht kontinuierliche Thoraxkompressionen und verringert somit die „no flow time“ (Unterbrechung der Thoraxkompression).
  - Die Beatmung erfolgt dann mit einer Frequenz von 10/min.

##### Der Fall.

Da außer Ihnen keine intubationserfahrene Person anwesend ist und sich die Masken-Beutel-Beatmung als effektiv erweist, behalten Sie diese zunächst bei. Somit können Sie sich zunächst einen besseren Überblick verschaffen und auf die Koordination des Teams und die Priorisierung von Aufgaben konzentrieren: Nach Etablierung eines Zugangs muss möglichst zeitnah die erste Adrenalindosis verabreicht werden. Nach 2 min folgt die nächste Rhythmuskontrolle: weiterhin PEA. Die Reanimation wird fortgesetzt, während Sie mögliche reversible Ursachen in Erwägung ziehen.

#### Welche reversiblen Ursachen müssen Sie berücksichtigen und wie können diese behandelt werden?

**Abb. 2:**
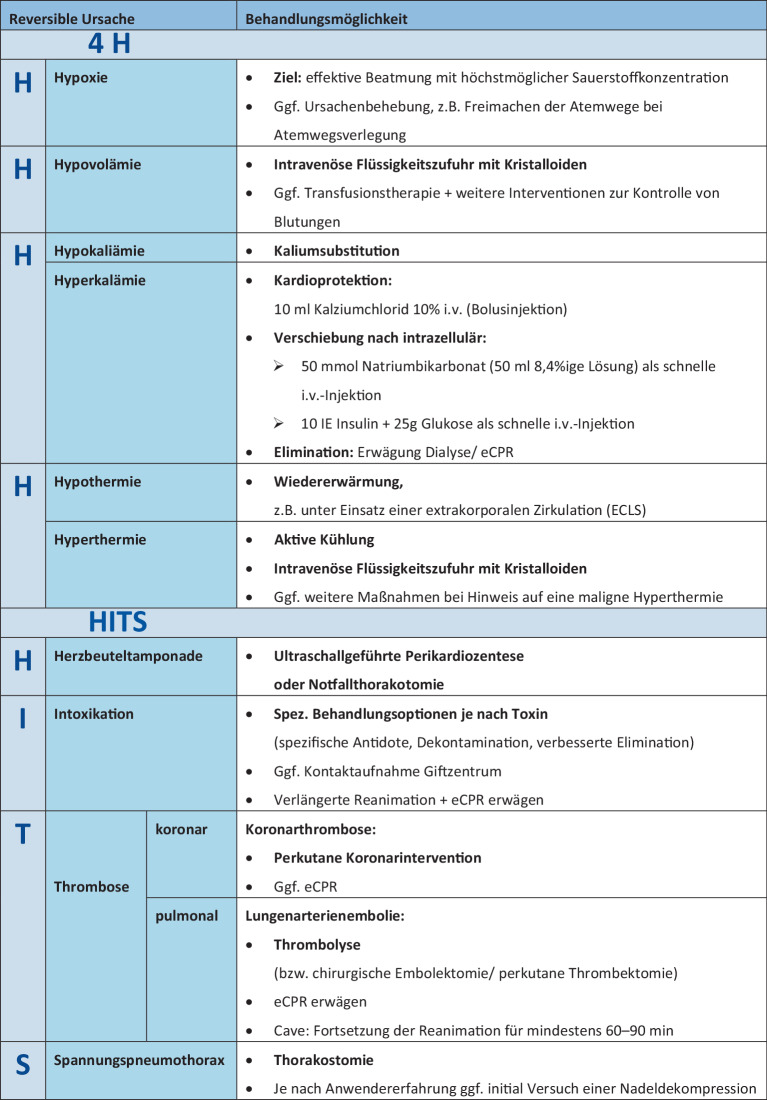
Reversible Ursachen (4 H und HITS) und deren Behandlungsmöglichkeiten nach den Leitlinien des European Resuscitation Council (ERC) 2025 [2]. *ECLS* „extracorporeal life support“, *eCPR* extrakorporale kardiopulmonale Reanimation

- Reversible Ursachen eines Kreislaufstillstands können bspw. mittels einer **Merkhilfe des ERC (4** **H und HITS)** strukturiert in Erwägung gezogen werden.
- Eine Übersicht über reversible Ursachen und deren Behandlungsmöglichkeiten gemäß den ERC-Leitlinien bietet Abb. 2.

##### Cave.

Der Einsatz der Sonographie kann hilfreich sein, um eine Herzbeuteltamponade oder einen Pneumothorax als reversible Ursache zu diagnostizieren. Hierbei sollte jedoch darauf geachtet werden, dass sich die „no flow time“ nicht verlängert und der Untersucher erfahren in der Anwendung der Notfallsonographie ist.

##### Merke.

- Nach Datenlage des deutschen Reanimationsregisters von 2024 [1] stellt ein **kardiales Geschehen weiterhin die Hauptursache innerklinischer Kreislaufstillstände** dar.
- Bei > 30 % der Patienten liegt als **zweithäufigste Ursache für den Kreislaufstillstand eine respiratorische Störung bzw. Hypoxie **vor, die mit einer schlechteren Prognose einhergeht.

##### Der Fall.

Die Befunde sprechen bei Ihrer Patientin für eine Hypoxie im Rahmen einer respiratorischen Dekompensation als Ursache des Kreislaufstillstands: Bei Aufnahme war die Patientin dyspnoisch und klagte über seit mehr als einer Woche bestehenden Husten und Auswurf. Die Röntgenuntersuchung des Thorax zeigte bilaterale bronchopneumonische Infiltrate. Hinweise auf eine Lungenarterienembolie oder einen Herzinfarkt bestanden nicht. Die Sauerstoffsättigung betrug bei Aufnahme trotz 6 l/min O_2_ 91 %, der Blutdruck 100/60 mm Hg und die Herzfrequenz 110/min; die Atemfrequenz wurde nicht dokumentiert.

#### Welche Maßnahme hätte den Kreislaufstillstand möglicherweise verhindern können?

- Durch die **Anwendung eines Track-trigger-Frühwarnsystems**, z. B. des National Early Warning Score (NEWS), können kritisch kranke Patienten und deren Zustandsverschlechterung frühzeitig erkannt werden.
- Durch die frühzeitige Einleitung entsprechender Maßnahmen kann ein Kreislaufstillstand ggf. verhindert werden.
- Dies setzt eine **entsprechende Schulung von Mitarbeitern** voraus.

##### Merke.

Die systematische Einschätzung von Patienten mit einem Scoring-System und entsprechende Verlaufskontrollen können einen Kreislaufstillstand ggf. verhindern. Die Anwendung innerklinischer Frühwarnsysteme wird deshalb dringend empfohlen [2].

##### Der Fall.

Um den mutmaßlichen Patientenwillen besser einschätzen zu können, fragen Sie das Stationsteam, ob bei Aufnahme der Einsatz von lebenserhaltenden Maßnahmen mit der Patientin besprochen wurde bzw. ob eine Patientenverfügung vorliegt.

#### Nach welchen Kriterien entscheiden Sie, ob die Reanimation fortgeführt oder beendet wird?

- Die Entscheidung über die Fortsetzung oder Beendigung einer Reanimation erfordert eine **ethisch reflektierte, patientenzentrierte und leitlinienkonforme Abwägung** [3].
- Praktisch ist die Umsetzung dieser Entscheidung äußerst komplex, da **oft unvollständige Informationen** vorliegen und **Entscheidungen nicht aufgrund einzelner Kriterien** getroffen werden sollten.
- Stark vereinfacht gesagt, müssen das medizinisch Machbare und der mutmaßliche Patientenwille in Einklang gebracht werden.

**Eindeutige Kriterien:**

- **Sichere Todeszeichen/nicht mit dem Leben vereinbare Verletzungen**
- **Dokumentierter Patientenwille**, z. B. im Rahmen einer gültigen Patientenverfügung oder einer aktuellen vorausschauenden Behandlungsplanung („advance care planning“), die sich gegen Reanimationsmaßnahmen ausspricht

**Kriterien mit Hinweis auf eine ungünstige Prognose:**

- Unbeobachteter Kreislaufstillstand
- Asystolie als initialer Rhythmus
- Reanimationsdauer ≥ 20 min
- Persistierend niedriges etCO_2_ < 10 mm Hg trotz adäquater Thoraxkompressionen
- Multimorbidität

##### Cave.

- Die genannten Kriterien weisen zwar auf eine ungünstige Prognose hin, sollten jedoch nicht isoliert in die Entscheidungsfindung einfließen.
- Das Behandlungsteam darf nicht eigene Vorstellungen von Lebensqualität unbewusst zum Maßstab machen. Entscheidend ist, welcher Gesundheitszustand für die Patientin selbst akzeptabel gewesen wäre.
- Pupillenreaktionen, Blutgasanalysen und Echokardiographie eignen sich nicht für eine valide Entscheidungsfindung.

##### Merke.

- Es sollte eine sorgfältige und patientenorientierte Abwägung zwischen medizinischer Indikation und Risiken für den Patienten erfolgen (Nutzen-Risiko-Verhältnis).
- Anhaltende Asystolie, eine Reanimationsdauer ≥ 20 min und das Fehlen reversibler Ursachen weisen auf eine ungünstige Prognose hin und können in Kombination die Entscheidungsfindung bzgl. der Beendigung einer Reanimation unterstützen.
- Die Entscheidung zur Beendigung der Reanimation liegt zwar in ärztlicher Verantwortung, die Entscheidungsfindung sollte jedoch gemeinsam im interprofessionellen Team erfolgen; daran sollte sich eine Nachbesprechung anschließen.

##### Der Fall.

Bevor Sie weitere Informationen einholen können, steht nach 2 min bereits die nächste Rhythmusanalyse an. Es zeigt sich eine Sinustachykardie von 105/min bei gut tastbarem Karotispuls. Nachdem Sie den Wiedereintritt eines Spontankreislaufs („return of spontaneous circulation“ [ROSC]) festgestellt haben, beginnen Sie mit der Postreanimationsbehandlung.

#### Welche Maßnahmen gehören zur strukturierten Postreanimationsbehandlung nach Wiedereintritt eines Spontankreislaufs?

Nach ROSC ist die Patientin weiterhin in einer vulnerablen Phase. Ziele der Postreanimationsbehandlung sind

- die **Stabilisierung der Vitalparameter**,
- die **Verhinderung sekundärer Schäden** sowie
- die **Klärung und Behandlung der zugrunde liegenden Ursache**.

Die Leitlinie des ERC und der European Society of Intensive Care Medicine (ESICM) von 2025 [4] empfiehlt ein strukturiertes Vorgehen entlang des **ABCDE-Schemas:**

**A – „airway“ (Atemweg):**

- **Atemwegssicherung** bei anhaltender Bewusstlosigkeit oder klinischer Indikation
- **Bestätigung der Tubuslage,** u. a. durch Kapnographie

**B – „breathing“ (Belüftung):**

- **Ziel: Normoxie/Normokapnie**:
  - Pulsoxymetrisch gemessene Sauerstoffsättigung (S_p_O_2_) 94–98 % (bzw. arterieller Sauerstoffpartialdruck [p_a_O_2_] 75–100 mmHg, etCO_2_ 35–45 mmHg).
- **Lungenprotektive Beatmung **(Atemzugvolumen: 6–8 ml/kg ideales Körpergewicht)

**C – „circulation“ (Kreislauf):**

- **12-Kanal-Elektrokardiogramm: **sofortige Koronarangiographie bei ST-Hebungs-Myokardinfarkt (STEMI) bzw. hochgradigem V. a. kardiale Genese des Kreislaufstillstands
- **Zuverlässiger i.v.-Zugang**
- **Hämodynamisches Monitoring:**
  - **Ziel des mittleren arteriellen Drucks:** > 60–65 mm Hg; kontinuierliche arterielle Blutdruckmessung.
    Ggf. **Monitoring Herzzeitvolumen** bei instabilen Patienten; **Verlaufskontrolle Laktat** (Ziel < 2,0–2,5 mmol/l), **Diurese** > 0,5 ml/kg pro h.
  - **Volumenersatztherapie **mit Kristalloiden bei Hypovolämie.
    **Ggf. Gabe von Vasopressoren/Inotropika **bei persistierender Hypotonie (**Noradrenalin** **=** **Mittel der Wahl, Dobutamin** ggf. bei persistierendem „low output“); **mechanische Kreislaufunterstützung bei persistierendem kardiogenem Schock**.
  - **Echokardiographie:** Beurteilung von globaler Pumpfunktion, Wandbewegungsstörungen etc.

##### Merke.

Ein kardiogener Schock mit reduziertem Herzzeitvolumen kann auch bei normotonem Blutdruck bestehen. Entscheidend ist die suffiziente Organperfusion, nicht allein der Druckwert. Hautdurchblutung, Kapillarfüllung und Vigilanz können klinische Hinweise geben.

**D – „disability“ (neurologischer Status):**

- **Blutzuckerkontrolle: **Ziel Normoglykämie
- **Ersteinschätzung neurologischer Status:** u. a. Glasgow Coma Scale (GCS), Pupillen, Motorik
- Regelmäßige neurologische Verlaufskontrollen und Aufwachversuche

##### Cave.

- **Aufwachversuch** auch bei Myoklonien!
- **Keine belastbare Prognose** **<** **72** **h!**
- **Kontrolle von Krampfanfällen:**
  - **Elektroenzephalographie (EEG)** zur Diagnostik und Therapieüberwachung.
  - **Erstlinientherapie:** antikonvulsive Therapie mit Levetiracetam oder Natriumvalproat.

##### Cave.

Keine prophylaktische Gabe von Antikonvulsiva!

**E – „exposure“ (Exploration):**

- **Temperaturmanagement:**
  - **Zieltemperatur:** ≤ 37,5 °C bei komatösen Patienten für 36–72 h.
  - **Keine aktive Erwärmung** komatöser Patienten mit milder Hypothermie (32–36 °C).
  - **Verwendung von Oberflächen- oder endovaskulärer Kühlung mit Feedbacksystem** im Falle einer Temperaturkontrolle.

**Das weitere intensivmedizinische Management umfasst u.** **a. Folgendes:**

- Ergänzende Diagnostik, z. B. Ganzkörper-CT bei nichtkardialer Ursache und entsprechenden Hinweisen
- Möglichst Oberkörperhochlagerung
- Frühzeitiger Beginn einer enteralen Ernährung
- Individuelle Antikoagulation
- Routinemäßige Stressulkusprophylaxe
- Verwendung kurz wirksamer Sedativa, tägliche Sedierungspausen
- **Keine** routinemäßige Gabe eines Muskelrelaxans!
- **Keine** routinemäßige prophylaktische Antibiotikatherapie!
- Nachbesprechung im Team und Rehabilitationseinleitung

##### Merke.

- Im Rahmen des **Postreanimationssyndroms** ist oft eine Kreislaufunterstützung notwendig.
- Das Postreanimationssyndrom umfasst
  - eine myokardiale Dysfunktion,
  - die systemische Ischämiereperfusionsreaktion und
  - neurologische Schädigungen.
- Das Postreanimationssyndrom ist die häufigste Ursache für Morbidität und Mortalität nach ROSC und erfordert ein strukturiertes, interdisziplinäres und engmaschiges Management.
- Eine zeitnahe **strukturierte Nachbesprechung** (Debriefing) kann zur verbesserten Teamdynamik und Prozessoptimierung beitragen und unterstützt die emotionale Verarbeitung.

##### Der Fall.

Bei der Untersuchung nach dem ABCDE-Schema zeigt sich die Patientin bradypnoisch und ohne Wachreaktion. Sie entscheiden sich daher, die Patientin noch vor Ort mittels Videolaryngoskopie zu intubieren, bevor sie zur weiteren Versorgung auf die Intensivstation verlegt wird.

#### Wie beurteilen und managen Sie die neurologische Prognose im weiteren Verlauf?

- Die neurologische Prognoseabschätzung nach Reanimation ist komplex und basiert auf einem **multimodalen, stufenweisen Ansatz** [4].
- Dabei ist entscheidend, dass die **endgültige Bewertung in der Regel frühestens 72** **h nach ROSC unter kontrollierten Bedingungen** (Normothermie, ohne sedierende Medikation) erfolgt.
- Elemente der neurologischen Prognoseabschätzung (Tab. 1):
  - **Klinisch-neurologische Untersuchung:** u. a. GCS, Hirnstammreflexe (Pupillenreaktion, Kornealreflex, Hustenreflex), Spontanmotorik vs. Strecksynergismen.
  - **Serumbiomarker:** neuronenspezifische Enolase (NSE) > 60 µg/l in zwei Proben mit 24 h Abstand mit schlechter Prognose assoziiert.
  - **Neurophysiologie** (z. B. EEG): generalisierte Suppression, Burst-suppression-Muster → schlechte Prognose.
  - **Bildgebung:** CT/Magnetresonanztomographie (MRT) zur Beurteilung globaler hypoxischer Hirnschädigung.
- Eine sehr schlechte Prognose, die zur Entscheidung über eine Therapielimitation führt, kann nur dann gestellt werden, wenn **mindestens zwei unabhängige Parameter mit hoher Spezifität für ein schlechtes neurologisches Outcome** (Cerebral Performance Category [CPC] 3–5) übereinstimmend pathologisch sind.

**Tab. 1:** Mögliche hochspezifische Prädiktoren (≥ 2 erforderlich)

Parameter	Kriterium für schlechte Prognose	Zeitpunkt
Klinik	Fehlen motorischer Reaktion auf Schmerzreiz (GCS‑M 1 oder 2) + beidseits fehlende Pupillen- und Kornealreflexe	≥ 72 h nach ROSC
Biomarker	NSE > 60 µg/l in zwei Proben mit 24 h Abstand (Tag 2 und 3)	48–72 h
EEG	Maligne Muster: z. B. generalisierte Suppression, „burst suppression“, Status epilepticus	24–72 h
SSEP	Bilateral fehlendes N20-Potenzial (somatosensibel evozierte Potenziale)	Ab 24 h
Bildgebung	CT: generalisierte hypoxische Schwellung/MRT: diffuse zytotoxische Schädigung der grauen Substanz	24–72 h

*CT* Computertomographie, *EEG* Elektroenzephalographie, *GCS‑M* Glasgow Coma Scale Motor Score (motorischer Score der Glasgow Coma Scale), *MRT* Magnetresonanztomographie, *NSE* neuronenspezifische Enolase, *ROSC* „return of spontaneous circulation“ (Wiedereintritt eines Spontankreislaufs), *SSEP* somatosensorisch evozierte Potenziale

##### Merke.

Mindestens zwei unabhängige Befunde mit hoher Spezifität (EEG, somatosensorisch evozierte Potenziale [SSEP], NSE, Klinik, Bildgebung) sind notwendig. Ein einzelner pathologischer Parameter reicht nicht zur sicheren Prognoseentscheidung!
